# Plasma Carotenoids and Biomarkers of Oxidative Stress in Patients with prior Head and Neck Cancer

**DOI:** 10.4137/bmi.s2192

**Published:** 2009-03-23

**Authors:** Kathryn J. Hughes, Susan T. Mayne, Jeffrey B. Blumberg, Judy D. Ribaya-Mercado, Elizabeth J. Johnson, Brenda Cartmel

**Affiliations:** 1 Yale School of Public Health, New Haven, Connecticut, U.S; 2 Yale Comprehensive Cancer Center, New Haven, Connecticut, U.S; 3 Jean Mayer USDA HNRCA at Tufts University, Boston, Massachusetts, U.S

**Keywords:** carotenoids, oxidative stress, isoprostanes, diet

## Abstract

Diets high in fruits and vegetables are generally believed protective against several chronic diseases. One suggested mechanism is a reduction in oxidative stress. The carotenoids, nutrients found in colored fruits and vegetables, possess antioxidant properties *in vitro*, but their role in humans is less well documented. The aim of this cross-sectional study was to explore the relationships between the most abundant plasma carotenoids (alpha-carotene, beta-carotene, lycopene, lutein, zeaxanthin and beta-cryptoxanthin), as well as grouped carotenoids (total xanthophylls, carotenes and carotenoids), and urinary excretion of the F_2_-isoprostanes (F_2_-IsoPs), stable and specific biomarkers of oxidative damage to lipids. Two F_2_-IsoP measures were utilized: total F_2_-IsoPs and 8-iso-PGF_2α_. The study population (N = 52) was drawn from a study among patients curatively treated for early-stage head and neck cancer. Unadjusted linear regression analyses revealed significant inverse associations between plasma lutein, total xanthophylls and both F_2_-IsoP measures at baseline. After control for potential confounders, all individual and grouped xanthophylls remained inversely associated with the F_2_-IsoP measures, but none of these associations achieved significance. The carotenes were not inversely associated with total F_2_-IsoPs or 8-iso-PGF_2a_ concentrations. The finding of consistent inverse associations between individual and grouped xanthophylls, but not individual and grouped carotenes, and F_2_-IsoPs is intriguing and warrants further investigation.

## Introduction

A number of studies support the hypothesis that diets high in fruits and vegetables protect against a variety of chronic diseases, including certain cancers and cardiovascular disease.^1^–^5^ It has long been suggested that carotenoids, which are found in fruits and vegetables, might be responsible for these protective effects as carotenoids have antioxidant properties *in vitro* and may act similarly in humans. Thus carotenoids may reduce oxidative stress, a state that has been associated with a number of diseases and which can be defined as “an imbalance between increased exposure to free radicals, principally derived from oxygen, and antioxidant defenses.”^6^

Carotenoids are fat-soluble pigments.^7^ Of the carotenoids that are quantifiable in human plasma, the most plentiful are alpha-carotene, beta-carotene, lycopene, lutein, zeaxanthin and beta-cryptoxanthin.^8^ *In vitro* studies have demonstrated that the carotenoids (particularly beta-carotene) are efficient scavengers of peroxyl radicals,^9^–^11^ which play a critical role in lipid peroxidation.^12^ The fact that carotenoids are lipid-soluble increases the plausibility that these nutrients may exert a protective effect against oxidative damage to lipids *in vivo*.^11^ Carotenoids can be classified into oxygenated carotenoids (e.g. lutein, zeaxanthin and beta-cryptoxanthin), known as xanthophylls, and hydrocarbon carotenoids (e.g. alpha-carotene, beta-carotene and lycopene), known as carotenes.^7^ Due to differences in chemical structure and polarity, carotenoids in these two classes attach at distinct locations of the cell membrane and thus may differentially influence the susceptibility of lipids to oxidation.^11^

Oxidative stress can be measured using various biomarkers of oxidative damage to DNA, proteins and lipids.^13^ In the early 1990s, a major advance in measuring oxidative damage of lipids was made with the discovery of the F_2_-Isoprostanes (F_2_-IsoPs) *in vivo*.^14^ The F_2_-IsoPs are stable and specific products of free radical catalyzed, nonenzymatic lipid peroxidation of arachidonic acid and detectable in all biological fluids.^14^,^15^ As F_2_-IsoP concentrations increase substantially in animal and human models of oxidative stress,^14^,^16^,^17^ these compounds appear to be reliable markers of free radical induced oxidative damage. One of the most abundant and commonly measured F_2_-IsoPs is 8-iso-PGF-_2α_, also known as 8-epi-PGF_2α_ or 15-F_2t_-IsoP^6^.

The evidence from studies examining the relationship between F_2_-IsoPs and fruit and vegetable intake or carotenoids is mixed. Several studies have documented a decrease in urinary F_2_-IsoPs with increased dietary intake of fruits and vegetables,^18^–^21^ while other studies have not observed this effect.^22^–^24^

The limited research that has been performed on the relationships between carotenoids, both the xanthophylls and carotenes, and F_2_-IsoPs in humans is not consistent. Cross-sectional studies have found significant inverse associations between plasma F_2_-IsoPs and plasma beta-carotene,^25^ as well as between urinary 8-iso-PGF_2α_ and plasma xanthophyll concentrations.^26^ A longitudinal study among children at high risk for developing type I diabetes demonstrated a significant inverse relationship between plasma alpha-carotene:total lipid ratio and urinary F_2_-IsoPs.^27^ Additionally, several trials involving consumption of foods rich in lycopene or a mixture of carotenoids and other nutrients found significant reductions in F_2_-IsoP concentrations after the intervention period.^28^–^30^ Decreased F_2_-IsoP concentrations were also observed after consumption of supplements containing beta-carotene alone or in combination with vitamin C and vitamin E.^31^,^32^ The trial with beta-carotene alone did not find a significant reduction, although this could be due to small sample size.^32^

However, other studies have not observed a protective effect of carotenoids against oxidative damage to lipids. Most of these null findings were obtained using measures other than F_2_-IsoPs as biomarkers of lipid peroxidation,^33^–^42^ and in one crossover trial, consumption of a tomato-based drink containing relatively small amounts of lycopene (5.7 mg) and beta-carotene (1 mg) among other nutrients for 26 days was not associated with a significant change in urinary 8-iso-PGF_2α_ excretion.^43^

Given the promising findings of *in vitro* studies and the limited epidemiologic studies using F_2_-IsoPs as outcomes, we conducted this analysis to explore the relationships between carotenoid and F_2_-IsoP concentrations in humans. We examined concentrations of the six major carotenoids found in human plasma, as well as grouped xanthophyll and carotene carotenoids and total carotenoids, in relation to concentrations of urinary F_2_-IsoPs using blood and urine samples collected on the same day. We hypothesized that inverse associations would be observed between the carotenoids and F_2_-IsoP measures. In addition, we hypothesized that a different relationship might be seen between the xanthophyll carotenoids and urinary F_2_-IsoPs compared to the carotenes and urinary F_2_-IsoPs.

## Materials and Methods

### Study population

The subjects in this analysis were drawn from 75 curatively treated patients who were identified as having been diagnosed with early-stage (*in situ*, Stage I or Stage II) cancer of the larynx, pharynx or oral cavity between January 1, 1997 and February 28, 2001 in Connecticut by the Rapid Case Ascertainment Shared Resource of the Yale Cancer Center (RCA). RCA acts as an agent of the Connecticut Tumor Registry (CTR).

The CTR is a population-based registry that has been part of the National Cancer Institute’s Surveillance, Epidemiology and End Results (SEER) Program since its inception in 1973. All procedures were approved by the Yale University School of Medicine Human Investigation Committee, the Connecticut Department of Public Health Human Investigation Committee and the participating hospitals. In the process of case ascertainment through RCA, certain data used in this study were obtained from the Connecticut Tumor Registry located in the Connecticut Department of Public Health. The authors assume full responsibility for analyses and interpretation of these data.

All patients gave informed consent to participate in a randomized dietary behavioral intervention study. The study has been described in detail elsewhere.^44^ Briefly, the study goal was to increase fruit and vegetable consumption in patients with early-stage, curatively treated head and neck cancer. A criterion for eligibility was that patients could not be taking more than 5 mg of supplemental beta-carotene per day at the time of recruitment. To be included in the present analysis, participants required baseline values for the two study outcomes, total F_2_-IsoPs and 8-iso-PGF_2α_, quantified from spot urine samples, and had to have assessment of baseline plasma carotenoids, quantified from blood samples.

### Baseline assessment

After obtaining written informed consent, baseline in-person assessments were made. The purpose of the baseline assessment was three-fold: to collect a spot urine sample to determine isoprostane excretion; to perform a blood-draw to ascertain plasma carotenoid concentrations; and to collect information on dietary, demographic and other factors of interest. Demographic variables assessed included age, sex, race, education, income and marital status. Further items on the questionnaire inquired about weight and height (to allow calculation of body mass index, BMI), alcohol consumption, smoking habits and use of beta-carotene dietary supplements. Dietary intake was assessed using the Fred Hutchinson Cancer Research Center Food Frequency Questionnaire, version 1992; the reference period was the prior month. Subjects were asked to abstain from fruit, vegetables, fruit juices and vitamin supplements for 6 hours prior to the visit so that carotenoid and F_2_-IsoP values would more accurately represent basal levels of these compounds *in vivo*.^45^

### Collection of urines and analysis for total F_2_-IsoPs and 8-iso-PGF_2__α_

All participants were asked to provide a spot urine sample at the baseline visit to allow determination of total F_2_-IsoP and 8-iso-PGF_2α_ concentrations. Following collection, the samples were kept cool during transportation to the laboratory, aliquoted and frozen at −70 °C until shipped on dry ice to the Jean Mayer USDA Human Nutrition Research Center on Aging (HNRCA) at Tufts University in Boston, Massachusetts for analysis. Total F_2_-IsoP and 8-iso-PGF-_2α_ concentrations were determined using high-performance liquid chromatography with gas chromatography/mass spectroscopy as described elsewhere.^46^ Final F_2_-IsoP concentrations were expressed as nanograms isoprostane per milliliter urine (ng/ml), based on prior work in our laboratory showing a significant positive association between smoking and 8-iso-PGF-_2α_ expressed per ml urine, but not between smoking and 8-iso-PGF-_2α_ standardized to creatinine.^32^

### Collection of blood samples and analysis for carotenoid concentrations

Interviewer-phlebotomists collected baseline blood samples from each subject in heparinized (green top) vacutainer tubes. After separation of the plasma portion of the blood, samples were stored at −70 °C until shipping and analysis at the HNRCA. Prior to the separation process, which occurred no more than 4 hours after the blood draw, samples were kept in a cold, dark location. Plasma samples were prepared for extraction using 200 μl of each sample and 0.5 ml 0.9% saline. Echinenone, in ethanol, was used as an internal standard. Two extraction steps were performed, first using 2 ml CHCl_3_:CH_3_OH (2:1, v:v) and then 3 ml hexane. Following each extraction step, the mixture was vortexed then centrifuged at 800 × g for 15 min at 4 °C. Additionally, the products obtained from the two extractions, the CHCl_3_ and hexane layers, respectively, were evaporated to dryness under nitrogen and combined. The mixture was then redissolved in 150 μl ethanol, vortexed and sonicated for 30 seconds. From each sample, a 50 μl aliquot was used for HPLC analysis. Before the procedure was begun, each HPLC solvent was passed through a 0.45 μm membrane filter and degassed and all carotenoid standards were stored at −70 °C.

The HPLC system consists of a 616 LC pump (Waters Corp., Milford, MA), Waters 717 plus autosampler (Waters Corp., Milford, MA), a C30 carotenoid column (3 μm, 150 × 4.6 mm, YMC, Wilmington, NC) and Waters 994 programmable photodiode array detector. The gradient reversed-phase HPLC procedure for plasma carotenoids has been described.^47^ The plasma data for this study were collected and analyzed using Millenium 32 Software (version 3.05.01, Windows NT, Waters Corp. 1998). Quantification of the carotenoids was accomplished by determining peak areas in the HPLC chromatograms calibrated against known amounts of standards. Concentrations were corrected for extraction and handling losses by monitoring the recovery of the internal standards. The lower limit of detection was 0.2 pmol for each carotenoid and only one subject fell below this level (for the alpha-carotene measure). For the purposes of this analysis, all three beta-carotene isomers were summed and all four lycopene isomers were summed to yield total beta-carotene and total lycopene measures.

### Data analysis

All analyses were performed using SAS, version 9.1 (SAS Institute, Cary, NC). Age, BMI, plasma concentrations of alpha-tocopherol (μg/dl) and dietary intakes of alcohol (g/day), vitamin C (mg/day) and fruits and vegetables (servings per day) were maintained as continuous variables. Sex, race (two levels), education (four levels), income (four levels), alcohol consumption (three levels), smoking status (two levels), marital status (two levels) and beta-carotene supplementation (three levels) were treated as categorical. For marital status, those who were never married, separated, widowed or divorced were combined into one category and those who were married or living as married comprised a second category. BMI was calculated by dividing each subject’s weight (in kg) by his or her height (in m^2^). To categorize alcohol consumption, each subject was classified into one of three categories: never/occasional drinker, former regular drinker and current regular drinker. The fruit and vegetable intake variable was constructed by summing servings of fruits and servings of vegetables consumed per day.

Median concentrations of the two study outcomes, total F_2_-IsoP and 8-iso-PGF_2α_, expressed as ng isoprostane per ml urine, were compared across levels of binary independent variables via Wilcoxon Rank Sum tests. Continuous predictors were divided at the median for the purposes of this analysis. Additionally, single-factor ANOVA was used to compare mean transformed concentrations of each outcome across levels of categorical independent variables with more than two levels. Log and square-root transformations were used to normalize the distributions of 8-iso-PGF_2α_ and total F_2_-IsoPs, respectively. Independent variables considered in Wilcoxon or ANOVA tests included demographic factors (age, sex, education, income and marital status), as well as alcohol consumption and smoking status, BMI, plasma alpha-tocopherol, dietary vitamin C, dietary fruit and vegetable intake, beta-carotene supplement use, the six plasma carotenoids of interest (alpha-carotene, beta-carotene, lycopene, lutein, zeaxanthin, and beta-cryptoxanthin), total xanthophyll concentration (sum of lutein, zeaxanthin and beta-cryptoxanthin plasma concentrations), total carotene concentration (sum of alpha-carotene, beta-carotene and lycopene plasma concentrations) and total plasma carotenoid concentration (sum of xanthophyll and carotene concentrations). Statistical significance was defined at the 0.05 level.

The relationships between concentrations of plasma carotenoids and total F_2_-IsoPs and 8-iso-PGF_2α_ were also explored via simple and multiple linear regression. All carotenoid measures were log-transformed prior to these analyses to normalize the distributions. The transformed F_2_-IsoP measures noted above were also used. Carotenoids considered were the six individual carotenoids, as well as total xanthophylls, total carotenes and total carotenoids.

Due to the strong correlation between individual carotenoids (e.g. alpha-carotene and beta-carotene rho > 0.7), separate regression models were generated for each carotenoid variable with each of the two F_2_-IsoP outcomes. Each multiple linear regression model included the following independent variables: smoking status, alcohol consumption, age, dietary vitamin C, plasma alpha-tocopherol, sex, BMI and education. These factors were selected as, based on a review of the literature, each was thought to be a potential confounder of the associations between concentrations of the carotenoids and the F_2_-IsoPs.

## Results

Of the 75 Fruit and Vegetable Trial participants, 52 had baseline measurements for total F_2_-IsoPs, 8-iso-PGF_2α_ and plasma carotenoids of interest and thus were eligible for inclusion in these analyses. Eight of the 75 participants in the intervention trial did not have baseline blood or urine samples, another six had blood samples, but not urine samples and another nine had urine samples, but not blood samples. Selected demographic and other characteristics of the study population are presented in Table 1. Of the 52 participants, 73% were male and almost all (50/52) were white, non-Hispanic. Seven of the participants were current smokers (mean pack-years = 83.3; sd ± 29.5) and 40 were former smokers (mean pack-years = 43.9; sd ± 26.7). The participants’ mean BMI was 26.2 kg/m^2^, indicating that participants on average tended to be overweight. The median plasma concentration of alpha-tocopherol (μmol/l) among participants was 28.0 (25th: 75th percentile 20.5:36.6). Of the 49 participants for whom prior treatment information was available, 47% of the participants had received radiation therapy alone, another 45% had had surgery alone and the remainder (8%) had received both treatments. The time between date of diagnosis and collection of study samples ranged from 6 to 33 months (25th percentile: 8 months, 50th percentile: 10 months, 75th percentile: 14 months). No patients were undergoing treatment at the time of biospecimen collection.

Six individual carotenoids were measured in plasma. Of these, lycopene and beta-carotene were the most abundant, whereas alpha-carotene and zeaxanthin were present in the lowest concentrations (Table 2).

In unadjusted analyses, we examined median urinary F_2_-IsoP concentrations by a number of demographic (Table 3) and plasma nutrient variables (Table 4). We found statistically significant inverse associations between plasma lutein and total F_2_-IsoPs in addition to total xanthophyll concentrations and total F_2_-IsoPs (Table 4), as well as a statistically significantly higher concentration of 8-iso-PGF_2α_ in current smokers compared to never and former smokers (Table 3). Smoking remained significantly positively associated with 8-iso-PGF_2α_ concentrations in the adjusted linear regression models for total carotenoids (beta = 0.7739, p = 0.047) and total carotenes (beta = 0.8188, p = 0.034). Although not significant, the direction of association with 8-iso-PGF_2α_ remained positive in the adjusted model for total xanthophylls (beta = 0.6014, p = 0.104). Based on the 10% rule for confounding, we found that alpha-tocopherol was a confounder of each carotenoid~isoprostane association and thus was retained in the models. However, alpha-tocopherol was not a significant predictor of either outcome at the 0.05 level.

In Table 5 we present unadjusted and adjusted associations between concentrations of the various carotenoid variables and total F_2_-IsoPs and 8-iso-PGF_2α_ from the linear regression models. Lutein and total xanthophylls were significantly inversely associated with urinary concentrations of both total F_2α_-IsoPs and 8-iso-PGF_2_ in the unadjusted models. After control for potential confounders, these associations remained inverse, but were attenuated and no longer achieved statistical significance. No statistically significant associations were seen between the individual or grouped carotenes and the F_2_-IsoP measures in the unadjusted or adjusted analyses.

## Discussion

The primary goal of this study was to explore the relationships between the six major carotenoids quantifiable in human plasma (alpha-carotene, beta-carotene, lycopene, lutein, zeaxanthin and beta-cryptoxanthin), as well as total xanthophylls, total carotenes and total carotenoids, and urinary isoprostane concentrations of both total F_2_-IsoPs and 8-iso-PGF_2α_. Adjusted analyses failed to show statistically significant associations between individual or grouped carotenoids and the two F_2_-IsoP measures.

While not always achieving statistical significance, the parameter estimates for xanthophylls in relation to biomarkers of lipid peroxidation were consistently negative, suggesting an inverse relation, which is consistent with previous findings in the literature. Two *in vitro* studies specifically examining the xanthophylls demonstrated a protective effect of lutein and zeaxanthin against UVB-induced lipid peroxidation.^48^,^49^ Additionally, a study in 37 female adult subjects found a significant inverse correlation between concurrent plasma xanthophyll (defined as the sum of plasma lutein and beta-cryptoxanthin concentrations) and urinary 8-iso-PGF_2α_ concentrations both before and after a two-week dietary intervention.^26^

Inverse associations were not observed between total carotenes and either F_2_-IsoP measure in this study. In the literature, evidence for an association between the carotenes and urinary isoprostanes is mixed, with some studies showing a significant protective effect^25^,^27^,^29^ and others finding no significant association.^43^

Our finding of a positive association between smoking and 8-iso-PGF_2α_ is consistent with previous findings in the literature. Two research groups found significantly greater urinary excretion of F_2α_-IsoP metabolites or 8-iso-PGF_2_ among smokers than age- and sex-matched nonsmokers.^16^,^17^ Further analyses conducted by one of the groups revealed a significant dose-response relationship between smoking exposure and urinary 8-iso-PGF_2α_ excretion, as well as a significant decline in urinary 8-iso-PGF_2α_ concentration after two or three weeks of smoking cessation.^17^

One of the major strengths of this study was the use of the gold standard measurement technique for the F_2_-IsoPs, gas chromatography/mass spectroscopy, GC/MS.^6^ One group investigated whether the less time-consuming and expensive ELISA can be used as a valid substitute for the GC/MS method, but weak agreement (Pearson correlation coefficient 0.51, 95% CI: 0.28–0.70 and weighted Kappa statistic 0.34) was found between these two methods.^50^ The use of high-performance liquid chromatography (HPLC), as opposed to the thin-layer chromatography method, to purify the F_2_-IsoPs prior to performing GC/MS in this study further enhanced the sensitivity and reproducibility of the assay.^46^ An additional strength of the study was the measurement of urinary as opposed to plasma F_2_-IsoPs, given that the former compounds are more stable and do not reflect pathways of 8-iso-PGF_2α_ formation other than free radical initiated peroxidation of arachidonic acid.^6^ Although urines were collected from a one-time sample during the day rather than totals over a 24-hour period, results from spot urines correlate well with those from 24-hour collections for the purpose of quantifying F_2_-IsoPs, in part because of the low variation in excretion of these compounds over the course of a day.^45^,^51^,^52^

Other strengths of this study included the ability to examine the association between concentrations of the F_2_-IsoPs and carotenoids taken at the same point in time (the baseline visit of the Fruit and Vegetable Trial) and the ability to control for many potential confounders. In addition to the carotenoid variable of interest, each adjusted model included the following predictors: smoking status, alcohol consumption, age, dietary vitamin C, plasma alpha-tocopherol, sex, BMI and education.

Limitations of this analysis include the small sample size (52 participants) and homogeneity of the study population, primarily white, formerly smoking males who had all been curatively treated for head and neck cancer, which could limit the generalizability of the results. The fact that such a low proportion of the sample was classified as current smokers was expected as these individuals had been diagnosed with and treated for head and neck cancer. However, the relative homogeneity of this sample in combination with a small sample size limited our power to detect significant inverse associations between several of the carotenoids and F_2_-IsoP outcomes. Also, as we performed multiple comparisons in these analyses, significant findings seen in the univariate comparisons could have arisen by chance.

Future research should continue to explore the precise nature of the relationship between the major carotenoids found in the plasma, in particular the xanthophylls given the promising findings reported in this study and others, and biomarkers of oxidative stress. As the F_2_-IsoPs are considered one of the most reliable markers of oxidative stress currently available,^6^ studies should continue to incorporate these markers but in larger and more heterogeneous samples, and ideally in the setting of a cohort study in which the predictive ability of these and other markers can be assessed.

## Figures and Tables

**Table 1:** Baseline characteristics of the study participants, N = 52.*

Characteristic	Mean ± SD or number of subjects (%)
Age (years)	62.6 ± 11.7
Sex	
Male	38 (73.1)
Female	14 (26.9)
Race	
White, non-Hispanic	50 (96.2)
Other	2 (3.9)
Education	
Less than high school	5 (9.6)
Some high school	25 (48.1)
Technical/business school or college	15 (28.9)
Graduate or professional school	7 (13.5)
Income (per yr)	
<41 K	16 (30.8)
41 K to <76 K	18 (34.6)
≥ 76 K	10 (19.2)
Unknown	8 (15.4)
Marital status	
Never/separated/widowed/divorced	14 (26.9)
Married/live as married	38 (73.1)
Alcohol consumption	
Never/occasional drinker	13 (25.0)
Former regular drinker	17 (32.7)
Current regular drinker	22 (42.3)
Smoking status	
Never/former smoker	45 (86.5)
Current smoker	7 (13.5)
BMI (kg/m^2^)	26.2 ± 4.9
Supplemental beta-carotene (IU)	
0	31 (59.6)
>0 and <5000	9 (17.3)
≥5000	12 (23.1)
Dietary alcohol intake (g/d)	13.1 ± 31.2
Dietary vitamin C (mg/d)	82.0 ± 57.5
Fruit/vegetable intake (servings/d)	2.9 ± 1.9

* Column percentages may not sum to 100 due to rounding.

**Table 2:** Median plasma carotenoid concentrations of the study subjects (N = 52).

Carotenoid (μmol/l)	Median (25th: 75th)*
Plasma alpha-carotene	0.048 (0.028:0.097)
Plasma beta-carotene	0.23 (0.10:0.45)
Plasma lutein	0.16 (0.11:0.22)
Plasma zeaxanthin	0.049 (0.033:0.066)
Plasma lycopene	0.30 (0.18:0.47)
Plasma beta-cryptoxanthin	0.071 (0.044:0.13)
Total plasma carotenoids	0.92 (0.63:1.51)

* Median values, as well as 25th and 75th quartiles are presented.

**Table 3:** Median and 25th: 75th percentile F_2_-isoprostane concentrations by demographic factors of interest.

Independent variable, divided at median if continuous	Total F_2_-IsoP, ng/ml urine	8-iso-PGF_2α_, ng/ml urine
Age (years)		
<64.5	10.53 (6.94: 14.20)	1.03 (0.69: 1.36)
≥64.5	12.97 (8.91: 16.79)	0.84 (0.44: 1.42)
Sex		
Male	10.74 (7.76: 15.53)	0.93 (0.65: 1.51)
Female	12.46 (9.27: 16.81)	0.99 (0.60: 1.35)
Education		
Less than HS	12.92 (10.83: 14.33)	1.51 (0.84: 1.52)
Some HS	13.64 (8.91: 16.79)	1.00 (0.68: 1.35)
Technical/business school or college	9.27 (5.46: 16.04)	0.85 (0.29: 1.62)
Graduate or professional school	9.31 (5.86: 12.32)	0.66 (0.37: 1.16)
Income (per yr)		
<41K	12.59 (9.18: 17.58)	1.04 (0.58: 1.47)
41K to <76K	10.27 (6.57: 15.08)	0.89 (0.65: 1.62)
≥76K	9.29 (6.94: 12.32)	0.88 (0.60: 1.10)
Unknown	15.44 (11.71: 18.66)	1.10 (0.63: 1.57)
Marital status		
Never/separated/widowed/divorced	12.46 (6.91: 16.81)	0.99 (0.37: 1.52)
Married/live as married	10.74 (8.11: 15.35)	0.93 (0.65: 1.36)
Alcohol consumption		
Never/occasional drinker	14.20 (10.64: 18.19)	1.25 (1.00: 1.36)
Former/regular drinker	12.26 (7.02: 15.08)	0.93 (0.65: 1.27)
Current/regular drinker	9.29 (6.57: 15.17)	0.81 (0.52: 1.62)
Smoking status		
Never/former	10.83 (7.76: 15.17)	0.87 (0.60: 1.27)
Current smoker	16.81 (8.25: 18.38)	1.62 (0.94: 2.10)*
BMI (kg/m^2^)		
<25.85	10.24 (6.57: 15.08)	0.85 (0.60: 1.08)
≥25.85	13.28 (9.26: 16.04)	1.21 (0.66: 1.52)
Supplemental beta-carotene (IU)		
0	10.83 (8.11: 16.79)	1.03 (0.78: 1.52)
>0 and <5000	15.17 (8.91: 16.04)	0.60 (0.29: 1.27)
≥5000	11.46 (7.39: 15.31)	0.77 (0.60: 1.24)
Dietary vitamin C (mg/d)		
<71.63	10.56 (6.94: 14.33)	1.02 (0.66: 1.36)
≥71.63	12.11 (8.91: 16.04)	0.88 (0.60: 1.51)
Fruit/vegetable intake (servings/d)		
<2.66	13.92 (9.09: 18.19)	1.04 (0.68: 1.42)
≥2.66	9.44 (7.02: 13.73)	0.84 (0.54: 1.35)

* Denotes significant difference (p < 0.05) in median or mean transformed F_2_-isoprostane concentration across categories of the independent variable, assessed via Wilcoxon tests for binary independent variables and via single-factor ANOVA for independent variables with >2 categories.

**Table 4:** Median and 25th: 75th percentile F_2_-isoprostane concentrations by plasma nutrient concentrations.

Independent variable, divided at median if continuous	Total F_2_-IsoP, ng/ml urine	8-iso-PGF_2α_, ng/ml urine
Alpha-carotene (μmol/l)		
<0.048	12.62 (9.09: 18.19)	0.93 (0.62: 1.63)
≥0.048	10.24 (6.57: 15.08)	0.98 (0.60: 1.27)
Beta-carotene (μmol/l)		
<0.23	12.62 (8.25: 18.37)	1.00 (0.69: 1.63)
≥0.23	10.24 (6.57: 14.33)	0.89 (0.44: 1.22)
Lutein (μmol/l)		
<0.164	14.27 (10.47: 18.19)	1.11 (0.78: 1.52)
≥0.164	9.29 (6.94: 13.02)*	0.84 (0.44: 1.22)
Zeaxanthin (μmol/l)		
<0.0492	13.33 (8.91: 16.04)	0.97 (0.65: 1.35)
≥0.0492	9.73 (6.91: 15.53)	0.86 (0.60: 1.42)
Lycopene (μmol/l)		
<0.30	12.62 (8.91: 16.81)	0.93 (0.60: 1.51)
≥0.30	10.27 (6.94: 15.35)	0.97 (0.62: 1.35)
Beta-cryptoxanthin (μmol/l)		
<0.0705	12.59 (8.11: 18.38)	1.04 (0.78: 1.51)
≥0.0705	10.71 (6.94: 15.35)	0.88 (0.54: 1.35)
Total xanthophylls (μmol/l)		
<0.3104	14.27 (9.09: 18.38)	1.04 (0.78: 1.52)
≥0.3104	9.44 (6.57: 13.02)*	0.88 (0.54: 1.25)
Total carotenes (μmol/l)		
<0.6337	12.62 (8.91: 18.37)	0.93 (0.66: 1.62)
≥0.6337	10.24 (6.57: 15.08)	0.97 (0.52: 1.25)
Total carotenoids (μmol/l)		
<0.918	12.62 (8.91: 18.37)	0.93 (0.66: 1.62)
≥0.918	10.24 (6.57: 14.20)	0.97 (0.52: 1.25)
Alpha-tocopherol (μmol/l)		
<28.00	12.59 (8.11: 18.19)	1.12 (0.84: 1.72)
≥28.00	10.65 (7.76: 15.17)	0.77 (0.54: 1.22)

*Denotes significant difference (p < 0.05) in median or mean transformed F_2_-isoprostane concentration across categories of the independent variable, assessed via Wilcoxon tests for binary independent variables and via single-factor ANOVA for independent variables with >2 categories.

**Table 5:** Unadjusted and adjusted† associations between concentrations of plasma carotenoids and urinary total F_2α_-IsoP and 8-iso-PGF_2_ .††

Carotenoid	Parameter estimate for unadjusted association with total F_2_-IsoP, in ng per ml urine (+/− standard error)*§	Parameter estimate for adjusted association with total F_2_-IsoP, in ng per ml urine (+/− standard error)*§	Parameter estimate for unadjusted association with 8-iso-PGF_2α_, in ng per ml urine (+/− standard error)*§	Parameter estimate for adjusted association with 8-iso-PGF_2α_, in ng per ml urine (+/− standard error)*§
Alpha-carotene	−0.1611 ± 0.1789	0.0204 ± 0.2017	−0.0851 ± 0.1454	0.0979 ± 0.1621
Beta-carotene	−0.0203 ± 0.1135	0.0753 ± 0.1308	−0.0630 ± 0.0914	0.0545 ± 0.1057
Lycopene	−0.0657 ± 0.1929	−0.0305 ± 0.2320	0.1336 ± 0.1551	0.1797 ± 0.1851
Lutein	−0.6569 ± 0.2240§	−0.4564 ± 0.2753	−0.4968 ± 0.1832§	−0.2075 ± 0.2275
Zeaxanthin	−0.4543 ± 0.2355	−0.2783 ± 0.2858	−0.3466 ± 0.1913	−0.0939 ± 0.2331
Beta-cryptoxanthin	−0.2761 ± 0.1661	−0.1574 + 0.2111	−0.1498 ± 0.1363	−0.0450 ± 0.1715
Total xanthophylls	−0.6629 ± 0.2316§	−0.4434 ± 0.2884	−0.4628 ± 0.1912*	−0.1765 ± 0.2381
Total carotenes	−0.0281 + 0.1589	0.0863 ± 0.1809	0.0161 ± 0.1285	0.1559 ± 0.1443
Total plasma carotenoids	−0.2315 ± 0.2032	−0.0398 ± 0.2417	−0.1079 ± 0.1658	0.1387 ± 0.1939

† Multivariate models for each carotenoid were adjusted for smoking, alcohol consumption, age, dietary vitamin C intake, plasma alpha-tocopherol concentration, sex, BMI and education.

†† Parameter estimates and associated p-values were obtained via simple and multiple linear regression, using transformed response and carotenoid variables.

* p < 0.05.

§ p < 0.01.
